# Accelerated long-term forgetting in presymptomatic autosomal dominant Alzheimer's disease: a cross-sectional study

**DOI:** 10.1016/S1474-4422(17)30434-9

**Published:** 2018-02

**Authors:** Philip S J Weston, Jennifer M Nicholas, Susie M D Henley, Yuying Liang, Kirsty Macpherson, Elizabeth Donnachie, Jonathan M Schott, Martin N Rossor, Sebastian J Crutch, Christopher R Butler, Adam Z Zeman, Nick C Fox

**Affiliations:** a Dementia Research Centre, University College London Institute of Neurology, London, UK; b London School of Hygiene & Tropical Medicine, London, UK; c Memory Research Group, Nuffield Department of Clinical Neurosciences, University of Oxford, Oxford, UK; d Cognitive Neurology Research Group, University of Exeter Medical School, Exeter, UK

## Abstract

**Background:**

Tests sensitive to presymptomatic changes in Alzheimer's disease could be valuable for clinical trials. Accelerated long-term forgetting—during which memory impairment becomes apparent over longer periods than usually assessed, despite normal performance on standard cognitive testing—has been identified in other temporal lobe disorders. We assessed whether accelerated long-term forgetting is a feature of presymptomatic autosomal dominant (familial) Alzheimer's disease, and whether there is an association between accelerated long-term forgetting and early subjective memory changes.

**Methods:**

This was a cross-sectional study at the Dementia Research Centre, University College London (London, UK). Participants were recruited from a cohort of autosomal dominant Alzheimer's disease families already involved in research at University College London, and had to have a parent known to be affected by an autosomal dominant Alzheimer's disease mutation, and not report any current symptoms of cognitive decline. Accelerated long-term forgetting of three tasks (list, story, and figure recall) was assessed by comparing 7-day recall with initial learning and 30-min recall. 7-day recognition was also assessed. Subjective memory was assessed using the Everyday Memory Questionnaire. The primary outcome measure for each task was the proportion of material retained at 30 min that was recalled 7 days later (ie, 7-day recall divided by 30-min recall). We used linear regression to compare accelerated long-term forgetting scores between mutation carriers and non-carriers (adjusting for age, IQ, and test set) and, for mutation carriers, to assess whether there was an association between accelerated long-term forgetting and estimated years to symptom onset (EYO). Spearman's correlation was used to examine the association between accelerated long-term forgetting and subjective memory scores.

**Findings:**

Between Feb 17, 2015 and March 30, 2016, we recruited 35 people. 21 participants were mutation carriers (mean EYO 7·2 years, SD 4·5). Across the three tasks, we detected no differences between carriers and non-carriers for initial learning or 30-min recall. The proportion of material recalled at 7 days was lower in carriers than non-carriers for list (estimated difference in mean for list recall −30·94 percentage points, 95% CI −45·16 to −16·73; p=0·0002), story (–20·10, −33·28 to −6·91; p=0·0048), and figure (–15·41, −26·88 to −3·93; p=0·012) recall. Accelerated long-term forgetting was greater in carriers nearer to their estimated age at onset (p≤0·01 for all three tests). Mutation carriers' 7-day recognition memory was also lower across all tasks (list [mean difference −5·80, 95% CI −9·96 to −2·47; p<0·01], story [–6·84, −10·94 to −3·37; p<0·01], and figure [–17·61, −27·68 to −7·72; p<0·01] recognition). Subjective memory scores were poorer in asymptomatic carriers compared with non-carriers (adjusted difference in means 7·88, 95% CI 1·36 to 14·41; p=0·016), and we found a correlation between accelerated long-term forgetting and subjective memory in mutation carriers.

**Interpretation:**

Accelerated long-term forgetting is an early presymptomatic feature of autosomal dominant Alzheimer's disease, which appears to pre-date other amnestic deficits and might underpin subjective memory complaints in Alzheimer's disease. Accelerated long-term forgetting testing might be useful in presymptomatic Alzheimer's disease trials.

**Funding:**

MRC, NIHR, Alzheimer's Research UK, Dementias Platform UK, Dunhill Medical Trust, ERUK, Great Western Research, Health Foundation, Patrick Berthoud Trust.

## Introduction

Pathological changes of Alzheimer's disease develop years before clinical symptoms.^1^, ^2^ The medial temporal lobe, which plays an important part in memory, is an early site of neurofibrillary tangle deposition and atrophy.^3^, ^4^, ^5^ Subtle cognitive impairment is likely to develop earlier than overt clinical symptoms, therefore cognitive measures sensitive to early changes would be valuable diagnostically and in presymptomatic trials.

Autosomal dominant (familial) Alzheimer's disease, due to mutations in presenilin 1 (*PSEN1*), presenilin 2 (*PSEN2*), or amyloid precursor protein (*APP*) genes, shares many features, both pathophysiologically and clinically, with the much more common sporadic form of the disease.^6^ Autosomal dominant Alzheimer's disease mutation carriers have relatively predictable ages at symptom onset based on family history,^7^ and therefore provide an opportunity to study presymptomatic cognitive change where time to symptom onset can be estimated.

**Research in context**

**Evidence before this study**

Accelerated long-term forgetting is a feature of temporal lobe epilepsy. We investigated whether accelerated long-term forgetting had been assessed in early Alzheimer's disease by searching PubMed for articles published between Jan 1, 1980, and April 11, 2016, with the terms “Alzheimer's disease” AND “long-term forgetting” OR “accelerated forgetting”. No articles that reported accelerated long-term forgetting in presymptomatic Alzheimer's disease were found. We then did a second PubMed search using the terms “Alzheimer's disease” AND “familial” OR “autosomal dominant” AND “presymptomatic” OR “preclinical” AND “cognitive” OR “memory” to identify studies of early cognitive change in autosomal dominant Alzheimer's disease. No langauge restrictions were used in either of the searches. The general pattern reported in these studies was of episodic memory decline evident up to 2–3 years before the expected onset of symptomatic disease, although one longitudinal study showed measurable decline only after the time of estimated onset. No studies assessing episodic memory had a retention interval of longer than 30 min. Visual short-term memory binding and dual task performance also declined in mutation carriers around 2 years before symptom onset. No studies were identified that showed earlier presymptomatic cognitive decline.

**Added value of this study**

To our knowledge, this study presents the first assessment of long-term forgetting in asymptomatic individuals who perform normally on conventional memory tests but have known Alzheimer's pathology. We found that differences in accelerated long-term forgetting were detectable in individuals who were on average 7 years away from predicted onset of symptomatic disease. We found that long-term retention of both verbal and visual material was affected, with impairment of recall and recognition. Accelerated long-term forgetting appears to pre-date other previously identified early Alzheimer's disease-related cognitive changes. Subtle increases in subjective cognitive concerns are also present several years before expected symptom onset.

**Implications of all the available evidence**

Our results add to existing evidence that episodic memory is impaired before symptom onset in individuals with Alzheimer's disease pathology. However, by assessing memory retention over a longer interval than is conventionally used, it is possible to detect verbal and visual memory deficits earlier than previously thought. Accelerated long-term forgetting appears to be one of the earliest detectable features of Alzheimer's disease-related cognitive decline. The results of this study extend our understanding of the temporal sequence of memory decline in Alzheimer's disease; it was previously possible to separate healthy ageing from Alzheimer's disease dementia and mild cognitive impairment by testing encoding and short interval retention. Assessment of accelerated long-term forgetting appears to provide a means of discriminating healthy ageing from asymptomatic individuals in the presymptomatic phase of Alzheimer's disease. Our findings of early, subtle subjective memory changes before overt symptoms in individuals with known Alzheimer's disease pathology, support research suggesting that subjective changes are predictive of future decline. The potential association between subjective memory change and accelerated long-term forgetting is consistent with previous epilepsy studies and, if confirmed, might explain the mechanism of early subjective change in Alzheimer's disease. Future work is required to define more precisely when deficits appear relative to actual symptom onset and to determine the use of accelerated long-term forgetting assessment in presymptomatic Alzheimer's disease trials.

Deficits in episodic memory are one of the earliest detectable cognitive abnormalities in Alzheimer's disease.^8^, ^9^, ^10^ However, there is a substantial delay between pathological change and measurable cognitive decline.^2^

Accelerated long-term forgetting is a form of memory impairment that has been described in patients with temporal lobe epilesy.^11^, ^12^ It refers to a process whereby new material appears to be encoded and retained normally over periods of up to 30 min—consistent with normal performance on standard memory tests—but is then forgotten at an abormally rapid rate over the following hours to weeks. Accelerated long-term forgetting might represent disruption in memory consolidation,^13^ which refers to gradual post-acquisition stabilisation of long-term memories, with the medial temporal lobe known to play an important part.^14^

Accelerated long-term forgetting was associated with subjective cognitive concerns in patients with epilepsy.^11^, ^12^ Such concerns might also be a preclinical harbinger of cognitive decline in Alzheimer's disease.^15^, ^16^, ^17^, ^18^

Accelerated long-term forgetting has recently been assessed in a mouse model of presymptomatic autosomal dominant Alzheimer's disease.^19^ Genetically modified mice had abnormal memory retention after 7 days, despite normal learning and retention over shorter intervals.

We hypothesised that accelerated long-term forgetting would also be a feature of presymptomatic autosomal dominant Alzheimer's disease in human beings, with mutation carriers forgetting more material over 7 days compared with non-carriers, despite similar learning and short interval (30 min) retention. Additionally, we investigated whether accelerated long-term forgetting is associated with subjective cognitive concerns.

## Methods

### Study design and participants

We did a cross-sectional study at the Dementia Research Centre, University College London (London, UK). Participants were recruited from families who were already actively involved (ie, consented to inclusion) in a long-running longitudinal study of autosomal dominant Alzheimer's disease at University College London. All participants, by having an affected parent, were at 50% risk of carrying a pathogenic autosomal dominant Alzheimer's disease mutation.^6^ Participants were also required to be asymptomatic, with neither they nor their informant reporting progressive cognitive symptoms. A further inclusion criterion was being aged older than 18 years. Participants were excluded from the study if they had substantial co-existing neurological or psychiatric disease. The study was approved by the local Research Ethics Committee and all participants provided written informed consent.

### Procedures

Genetic testing was done to establish mutation status and results were provided only to statisticians, ensuring that participants and clinicians assessing them remained blind to genetic status. All individuals identified a close informant who was interviewed separately to gain a collateral history. Estimated years to symptom onset were calculated by subtracting the participant's age at testing from the age at which their affected parent first developed progressive cognitive symptoms.

Participants underwent neurological examination and assessment using the mini mental state examination and the Clinical Dementia Rating scale,^20^ which incorporates information from both participant and informant on day-to-day cognition. Neuropsychological testing included measures of general intellectual functioning (Wechsler Abbreviated Scale of Intelligence [WASI]),^21^ verbal and visual recognition memory (Recognition Memory Test for words and faces),^22^ and paired associate learning (Camden paired associate learning test).^23^ Depression and anxiety were assessed using the Hospital Anxiety and Depression Scale (HADS).^24^ Subjective memory was assessed using the Everyday Memory Questionnaire (EMQ),^25^ scored between 0 and 90, with higher scores indicating greater concerns.

Long-term forgetting was assessed using test materials from the Adult Memory and Information Processing Battery.^26^ Assesment was 1–3 months after background clinical data collection. This 2-month window was allowed because of practical considerations that meant it was not always possible for every participant to return for assesment after exactly the same interval. A minimum 1-month gap was left between neuropsychological testing and long-term forgetting assessment so that participants did not confuse material from the two types of test. Participants were assessed on three tests: learning and recall of a 15-item word list, a short story, and a complex visual figure. For the word list, participants had to learn the material to a minimum required accuracy of 80% over a minimum of four and maximum of ten trials. For the story, the minimum required accuracy was 80%, over a minimum of two and maximum of ten trials. For the figure, participants were first asked to copy the figure as accurately as possible on to a separate piece of paper; the figure and copy were then removed and participants were asked to draw the figure again from memory. Participants were then tested on free recall of the word list, story, and figure 30 min after presentation of the last learning trial for each test. All three tests (list, story, and figure) were marked by the same assessor (PSJW) using validated marking criteria.^26^

There were two versions of each test. Participants were randomly assigned to either set one or set two (a random number was generated [either 1 or 2] before testing each participant, which was then used to decide the set), except for families in which two members were participating, in which case the second participant in a family was always assessed on a different version from the first participant. Participants were requested not to discuss the tests with other participants.

Following assessment, participants were given an envelope (appendix), which they were asked not to open until told to do so during a telephone call 7 days later. Participants were not told that the memory tests would be repeated. At the 7-day telephone call, participants' free recall of the test materials was reassessed. For the figure, they were asked to draw the figure on a blank piece of paper included in the envelope, and for the verbal tests recall was done orally. Forced-choice recognition memory was then assessed. For the word list, participants were read 15 semantically unrelated word pairs, and asked to identify which of each pair was in the original list. A similar three-alternative forced-choice response procedure was done for 12 separate aspects of the story. For the figure, participants were asked to view a sheet (contained in a separate envelope) showing four sets of three similar illustrations and were asked to mark which of each set of three exactly matched part of the figure. Figure assessments were returned in a stamped addressed envelope.

### Outcomes

The primary outcome measure of interest was the proportion of material (list, story, and figure) retained at 30 min that was recalled 7 days later (ie, 7-day recall divided by 30-min recall). This approach, used in previous studies in patients with epilepsy,^27^ allows comparison of group differences at 7 days, adjusting within each individual for short interval (30-min) forgetting. Secondary outcomes were 7-day recall and recognition scores for each of the three tasks. The only other secondary outcome was subjective memory score, although this was separate from the long-term forgetting analysis.

### Statistical analysis

Scores on standard cognitive tests, HADS, EMQ, and accelerated long-term forgetting initial learning (score on the final learning trial), number of trials to criterion, 30-min recall, 7-day recall, 7-day score divided by 30-min score, and 7-day recognition were compared between mutation carriers and non-carriers using linear regression with robust SEs to account for clustering of participants within families. Linear regression was used to examine the association between 7-day score divided by 30-min score on each accelerated long-term forgetting test and estimated years to symptom onset (EYO), with robust SEs to account for clustering of participants within families. If plots of model residuals indicated a departure from the assumption of normal distribution with constant variance, statistical inference was based on non-parametric bias-corrected and accelerated 95% CIs and 99% CIs from 10 000 bootstrap replications clustered on family.

Comparison of HADS, mini mental state examination, Recognition Memory Test faces, Recognition Memory Test words, Camden paired associated learning test, and EMQ was adjusted for age and intelligence quotient (IQ; WASI total). Comparison of WASI performance IQ and WASI verbal IQ was unadjusted, as these measures were corrected for age. Comparison of groups with regards to accelerated long-term forgetting scores was adjusted for age, IQ, and test set.

Non-parametric receiver operating characteristic (ROC) curves were used to quantify the ability of the 7-day score divided by the 30-min score on each of the accelerated long-term forgetting tests to discriminate between carriers and non-carriers. Areas under the curves (AUCs) were calculated and cutoffs, specificities, and sensitivities reported.

The association between EMQ and 7-day score divided by 30-min score on each accelerated long-term forgetting test was assessed using the non-parametric Spearman correlation coefficient, with inference based on non-parametric bias-corrected and accelerated 95% and 99% CIs from 10 000 bootstrap replications clustered on family. For analyses involving EMQ, only participants who were unaware of their mutation status were included, as knowledge of status might bias an individual's perception of their memory.

Results were interpreted taking into consideration that a Bonferroni correction for use of three accelerated long-term forgetting tests (list, story, figure) would require p<0·017 (ie, 0·05 divided by 3) for formal statistical significance.

For the four figure recall and recognition assessment documents that were not returned, these data were assumed to be missing entirely at random. Data were not imputed for missing cases.

All statisitcal analyses were done using Stata version 14.0 or later.

### Role of the funding source

The funders of the study had no role in study design, data collection, data analysis, data interpretation, or writing of the report. The corresponding author had full access to all the data in the study and had final responsibility for the decision to submit for publication.

## Results

We recruited 35 asymptomatic individuals from 19 autosomal dominant Alzheimer's disease families between Feb 17, 2015, and March 30, 2016. Nine participants (five mutation-positive, four mutation-negative) had previously chosen to have clinical predictive genetic testing, and so were aware of their genetic status. 21 (60%) of 35 participants were mutation carriers. All participants scored 0 for both global Clinical Dementia Rating scale and Clinical Dementia Rating scale Sum of Boxes. Carriers and non-carriers were well matched for age, sex, and years in education (table 1). All participants were right-handed. Carriers were on average 7·2 years (SD 4·5) from estimated symptom onset. Scores for mutation carriers and non-carriers did not differ for mood and for most conventional tests of memory and global intelligence, although carriers had higher recognition memory for words (adjusted estimated difference in means 2·2, 95% CI 1·0–3·6; p<0·01 and non-carriers had slightly higher performance IQ (8·2, 1·0–15·4; p=0·027; table 2).

**Table 1:** Participant characteristics

		**Mutation carriers (n=21)**	**Non-carriers (n=14)**
Sex			
	Male	11 (52%)	6 (43%)
	Female	10 (48%)	8 (57%)
Age (years)		38·0 (5·9)	39·2 (7·9)
Estimated years to symptom onset		7·2 (4·5)	NA
Years of education		13·8 (2·5)	14·4 (2·2)

Data are n (%) or mean (SD). NA=not applicable.

**Table 2:** Mood assessment and standard tests of intelligence and memory results

	**Mutation carriers**	**Non-carriers**	**p value**
HADS anxiety score (out of 21)	6·0 (3·0–9·0)	4·5 (4·0–7·0)	0·47
HADS depression score (out of 21)	2·0 (0·0–2·0)	1·0 (0·0–2·0)	0·59
Mini mental state examination (out of 30)	29·0 (29·0–30·0)	29·5 (29·0–30·0)	>0·05
WASI verbal IQ	106 (91–108)	103 (95–103)	0·37
WASI performance IQ	104 (100–116)	119 (106–121)	0·03
Recognition Memory Test words (out of 50)	49·0 (49·0–50·0)	48·0 (46·0–50·0)	<0·05
Recognition Memory Test faces (out of 50)	45·0 (43·0–48·0)	45·5 (44·0–47·0)	>0·05
Camden paired associate learning (out of 24)	19·0 (16·0–22·0)	19·0 (17·0–22·0)	>0·05

Data are median (IQR). p values are shown for variables that satisfied the assumptions of the linear regression model. For other variables, parametric assumptions were not met and therefore p<0·05 or p>0·05 was inferred from bootstrapped 95% CIs. For HADS anxiety, HADS depression, mini mental state examination, Recognition Memory Test faces, Recognition Memory Test words, and Camden paired associate learning, p values reflect group differences after adjusting for age (years) and IQ (WASI total score). Analysis of WASI performance IQ and WASI verbal IQ was unadjusted, as these measures were corrected for age. HADS=Hospital Anxiety and Depression Scale. WASI=Wechsler Abbreviated Scale of Intelligence.

**Figure 1:**
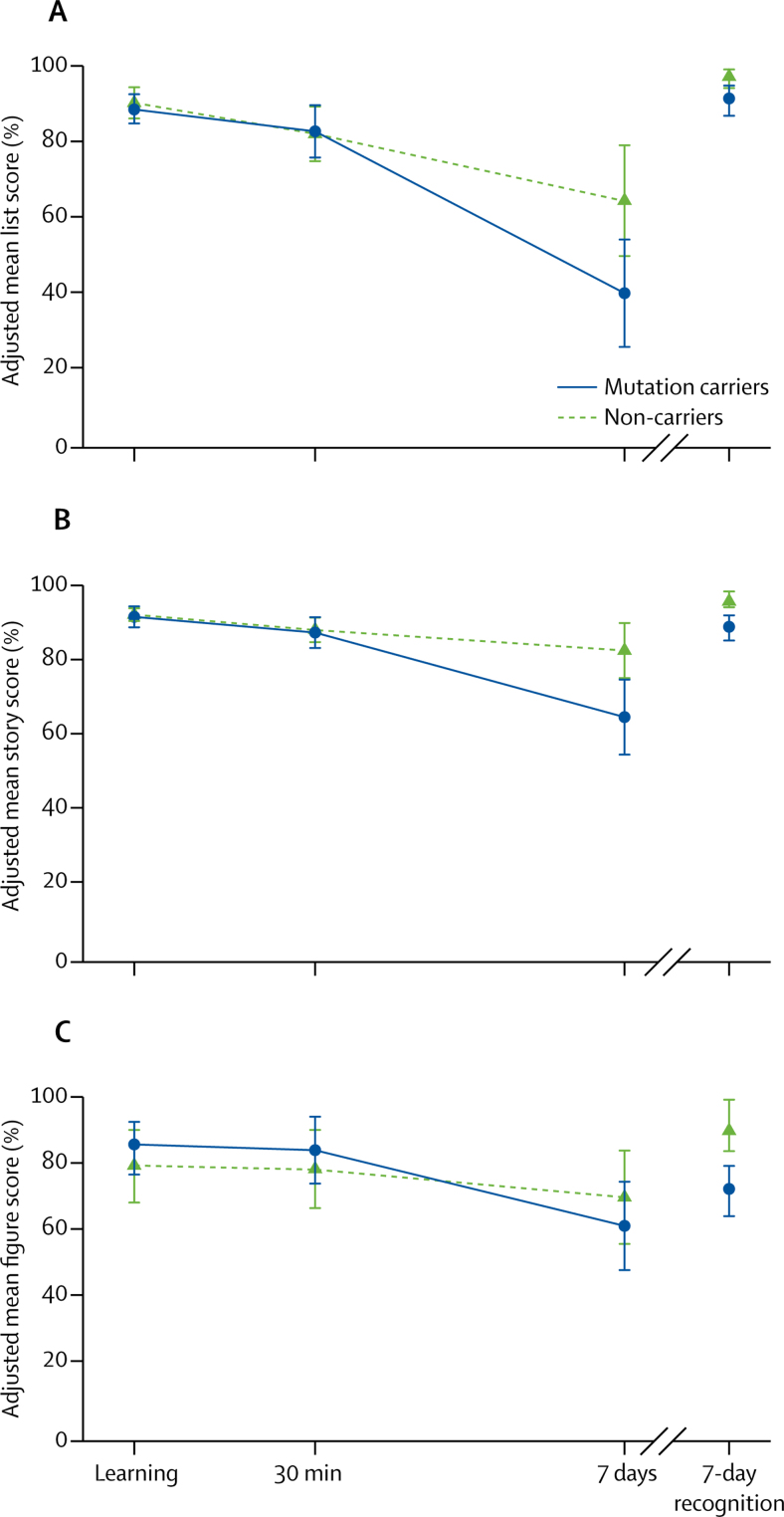
Long-term forgetting assessments Scores for (A) word list, (B) story, and (C) figure were adjusted for age, intelligence quotient (WASI total score), and test set. Robust SEs accounted for within-family clustering. WASI=Wechsler Abbreviated Scale of Intelligence.

All participants learned the word list and story to the required 80% accuracy within the allowed number of trials. Across all three accelerated long-term forgetting tests, adjusting for age, IQ, and test set, and accounting for clustering within families, the ability of mutation carriers to learn the material did not differ from non-carriers, both in terms of initial score and in the number of learning trials (table 3). Recall score at 30 min for all three tasks did not differ between groups (table 3, figure 1). All participants completed all assessments. However, four participants failed to return the 7-day figure recall and recognition assessment documents, so these could not be included in the analysis of these measures.

**Table 3:** Long-term forgetting assessment results

	**Mutation carriers**	**Non-carriers**	**p value**	**Difference in mean**	**95% CI**
**Learning**					
List learning trials	5·0 (4·0–6·0)	4·0 (4·0–6·0)	>0·05	−0·06	−1·01 to 0·97
Story learning trials	3·0 (2·0–3·0)	2·0 (2·0–3·0)	>0·05	0·07	−0·50 to 0·82
List learning score	80·0% (80·0–86·7)	86·7% (80·0–93·3)	>0·05	−1·77	−7·01 to 2·49
Story learning score	87·5% (83·3–91·1)	90·5% (87·5–94·6)	0·67	−0·60	−3·50 to 2·30
Figure learning score	100·0% (97·4–100·0)	100·0% (99·3–100·0)	>0·05	6·39	−4·00 to 15·21
**30-min retention**					
List 30-min recall	80·0% (73·3–80·0)	80·0% (73·3–86·7)	0·85	0·66	−6·52 to 7·84
Story 30-min recall	83·3% (80·0–87·5)	87·9% (80·4–91·1)	0·76	−0·74	−5·77 to 4·29
Figure 30-min recall	92·5% (72·4–97·4)	88·4% (72·4–96·7)	0·34	5·72	−6·55 to 17·98
**7-day retention (seconday outcomes)**					
7-day list recall	26·7% (20·0–46·7)	56·7% (46·7–66·7)	0·0021	−24·55	−39·01 to −10·10
7-day story recall	56·7% (50·0–68·3)	79·3% (71·4–83·9)	0·0031	−17·97	−29·06 to −6·88
7-day figure recall	59·2% (48·8–73·7)	74·3% (62·5–83·2)	0·22	−8·67	−22·91 to 5·57
7-day list recognition	93·3% (86·7–100·0)	100·0% (100·0–100·0)	<0·01	−5·80	−9·96 to −2·47
7-day story recognition	91·7% (91·7–100·0)	100·0% (100·0–100·0)	<0·01	−6·84	−10·94 to −3·37
7-day figure recognition	75·0% (75·0–100·0)	100·0% (100·0–100·0)	<0·01	−17·61	−27·68 to −7·72
**7-day retention (primary outcome)**					
List 7-day recall divided by 30-min recall	36·4 (30·0–58·3)	71·8 (55·6–81·8)	0·0002	−30·94	−45·16 to −16·73
Story 7-day recall divided by 30-min recall	66·7 (58·8–81·3)	92·2 (81·1–95·7)	0·0048	−20·10	−33·28 to −6·91
Figure 7-day recall divided by 30-min recall	70·3 (63·2–82·1)	87·6 (76·9–96·4)	0·012	−15·41	−26·88 to −3·93

Data are uncorrected group medians (IQR). Exact p values are shown for variables that satisfied the assumptions of the linear regression model. For other variables, parametric assumptions were not met and therefore p<0·05 or p>0·05 and p<0·01 or p>0·01 was inferred from bootstrapped 95% and 99% CIs. Differences in means, 95% CIs, and p values are adjusted for age, intelligence quotient (Wechsler Abbreviated Scale of Intelligence total score), and test set. Robust standard errors accounted for clustering of participants within families. Secondary outcome measures for each of the three tests were 7-day recall and recognition scores, with the primary outcome measure being 7-day recall divided by 30-min recall. For the list and story, learning score refers to the score from the final learning trial.

For 7-day recall scores divided by 30-min recall scores, mutation carriers did worse than non-carriers for list (adjusted difference in means −30·94 percentage points, 95% CI −45·16 to −16·73; p=0·0002), story (–20·10, −33·28 to −6·91; p=0·0048) and figure (–15·41, −26·88 to −3·93; p=0·012) recall.

The poorer retention of material at 7 days in mutation carriers was also seen in lower mean 7-day recall scores for list and story categories than in non-carriers, but not for figure (table 3). Mutation carriers also did worse than non-carriers on 7-day list, story, and figure recognition (table 3).

**Figure 2:**
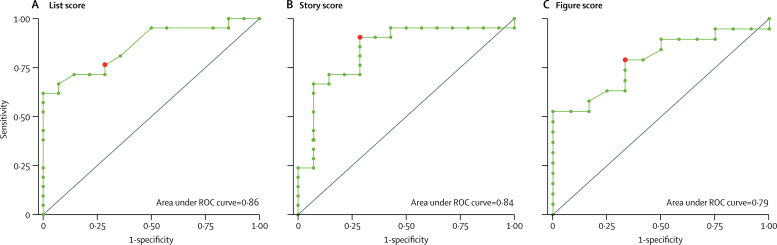
ROC curves for accelerated long-term forgetting testing as a discriminator between presymptomatic mutation carriers and non-carriers ROC curves are shown for 7-day score divided by 30-min score for (A) list, (B) story, and (C) figure scores. Red dots indicate the point on each curve for the proposed optimum cutoffs. ROC=receiver operating characteristic.

ROC analysis for discrimination between mutation carriers and non-carriers for 7-day score divided by 30-min score revealed an AUC of 0·86 (95% CI 0·66–0·95) for list, 0·84 (0·62–0·96) for story, and 0·79 (0·53–0·93) for figure scores (figure 2). A cutoff of less than 60% on list long-term retention gave 76% sensitivity and 71% specificity for mutation carriers versus non-carriers. A cutoff of less than 88% for story recall gave 90% sensitivity and 71% specificity. A cutoff of less than 75% for figure recall gave 79% sensitivity and 67% specificity.

**Figure 3:**
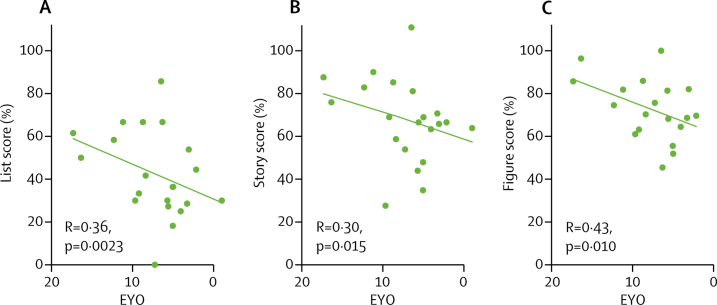
Scatter plots for EYO against long-term recall scores in mutation carriers (A) Word list, (B) story, and (C) figure. EYO=estimated years to onset.

There was an association between long-term forgetting and EYO for list, story, and figure scores, with the severity of accelerated long-term forgetting increasing with proximity to predicted symptom onset (figure 3).

**Figure 4:**
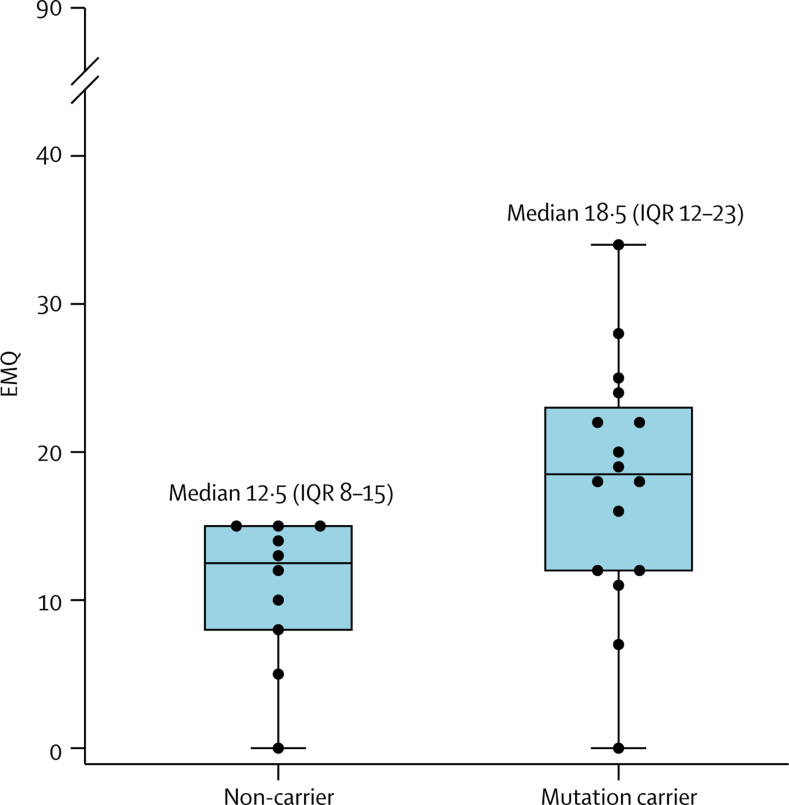
Box and whisker plots, with individual data points superimposed for EMQ score Boxes show 25–75th percentile, lines show median, and whiskers show range of EMQ scores. The score is out of a maximum of 90, with higher scores indicating greater subjective cognitive concerns. Only individuals who did not know their genetic status were included. EMQ=Everyday Memory Questionnaire.

**Figure 5:**
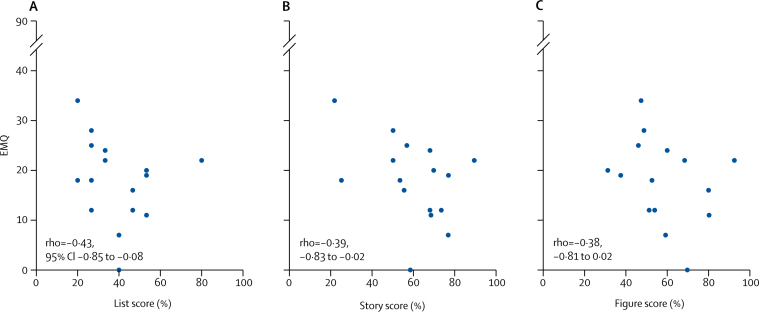
EMQ score against long-term recall score in mutation carriers Comparison of EMQ score with long-term forgetting score (7-day recall divided by 30-min recall) for (A) word list, (B) story, and (C) figure. Only the 16 mutation carriers who did not know their genetic status were included. EMQ=Everyday Memory Questionnaire.

Neither mutation carriers (median 18·5 [IQR 12–23] of 90 maximum) or non-carriers (12·5 [8–15] of maximum of 90) had high EMQ scores (having excluded participants who knew their genetic status). However, mutation carriers had more subjective memory concerns than did non-carriers (adjusted difference in means 7·88, 95% CI 1·36–14·41; p=0·016) (figure 4). In mutation carriers, an association existed between higher EMQ and poorer long-term retention (7-day recall score divided by 30-min recall score), which reached statistical significance for both list and story, but not for figure (figure 5). There was no evidence of an association between EMQ score and performance on standard cognitive tests.

## Discussion

We found that accelerated long-term forgetting was a feature of presymptomatic autosomal dominant Alzheimer's disease in a group of individuals who were on average 7 years from estimated symptom onset. Despite similar learning and ability to recall over a short interval, reassessment 7 days later showed that presymptomatic mutation carriers had forgotten more than had non-carriers. This finding was consistent for recall and recognition memory, and for verbal and non-verbal material. Among mutation carriers, severity of accelerated long-term forgetting increased with proximity to estimated symptom onset.

By contrast, mutation carriers showed no impairment in performance on conventional memory tests, which included both recognition memory for words and faces and paired associate learning. This finding suggests that differences in long-term forgetting are unlikely to be due to problems with encoding or early retention, but rather are caused by impairment of long-term memory consolidation. Although impairment of episodic memory is recognised as a central component of the Alzheimer's disease phenotype, relatively little is known about how long-term consolidation of memory is affected. Some studies have investigated potential consolidation deficits in early Alzheimer's disease,^28^ but it is difficult to measure long-term retention of memory reliably and compare a disease group with controls, unless the groups are equal for initial encoding and short interval retention, which in these studies was not the case.^29^, ^30^ By recruiting a presymptomatic Alzheimer's disease cohort, and testing retention over a longer period than in standard neuropsychological assessment, we showed that impairment of long-term retention is an early amnestic feature of Alzheimer's disease, and might be one of the earliest features of Alzheimer's disease-related cognitive decline.

A previous study^8^ of autosomal dominant Alzheimer's disease tested recognition memory over a more conventional interval of about 5 min and identified presymptomatic memory changes, but only 1–2 years before symptom onset. Other studies^31^, ^32^, ^33^ of presymptomatic carriers (from the *PSEN1* Colombian kindred) showed reduced cognitive performance in several domains, including visual short-term memory binding, dual task performance, and lexical-semantic processing; however, participants were closer to expected symptom onset than were those in our study. Another study^34^ of the same cohort found that earlier decline could be identified by using a composite score comprising multiple tests. A similar composite cognitive test approach, which included paired associate learning, delayed recall, naming, and digit-symbol substitution (an assessment of short-term memory and processing speed) identified decline in individuals in the late stage of presymptomatic sporadic Alzheimer's disease.^35^

We also found group differences in recognition memory at 7 days. Impaired recognition, which implies a so-called storage deficit rather than an isolated retrieval deficit, is consistent with pathology primarily affecting the medial temporal lobe,^36^ a brain region affected early in Alzheimer's disease.

ROC analysis indicated that accelerated long-term forgetting assessments might provide a means of discriminating between presymptomatic Alzheimer's disease and healthy ageing, with discriminatory power being stronger for verbal tests (list and story) than for the figure test. However, the sample size of the current study limits the precision with which AUCs can be estimated. Moreover, specificity values for our proposed cutoffs are suboptimal, and further work is needed to determine the most suitable cutoffs.

Our findings advance existing understanding of the stages of memory decline in Alzheimer's disease. Previous studies^37^ have reported discrimination of Alzheimer's disease-dementia from Alzheimer's disease mild cognitive impairment based on initial encoding of memory, which has been found to be impaired in the dementia stage but not in mild cognitive impairment. Similarly, Alzheimer's disease mild cognitive impairment can be separated from presymptomatic disease and healthy ageing based on short-term retention (eg, over 30 min), which is impaired only in Alzheimer's disease mild cognitive impairment. Our results suggest that incorporating tests of long-term (eg, 7-day) retention might allow individuals with presymptomatic Alzheimer's disease (but without conventional deficits) to be distinguished from healthy ageing (table 4).

**Table 4:** Proposed stages of progressive memory impairment in Alzheimer's disease, relative to healthy ageing

	**Memory encoding**	**Early retention**	**Long-term retention**
Alzheimer's disease dementia	Reduced	Reduced	Reduced
Mild cognitive impairment	Normal/unchanged	Reduced	Reduced
Presymptomatic Alzheimer's disease	Normal/unchanged	Normal/unchanged	Reduced
Healthy ageing	Normal/unchanged	Normal/unchanged	Normal/unchanged

Previous studies have established differences in memory function between Alzheimer's disease dementia, Alzheimer's disease mild cognitive impairment, and healthy ageing, based on tests of memory encoding and retention over a short interval. The results of our study allow us to propose a third column, for long-term retention, which shows impairment several years before onset of noticeable symptoms.

Other than accelerated long-term forgetting, the only group difference where carriers performed worse than non-carriers on cognitive testing was for performance IQ. This finding is consistent with previous findings in autosomal dominant Alzheimer's disease that, along with episodic memory, reduced performance IQ is one of the earliest cognitive changes.^8^ However, the proportional difference in performance IQ between groups was much smaller than for accelerated long-term forgetting. As we adjusted for WASI score in all regression analyses, it is unlikely that the difference in performance IQ influenced the main study findings.

Despite reporting no overall concerns about their memory and being unaware of their genetic status, we found that presymptomatic mutation carriers had worse subjective memory scores than non-carriers, which supports emerging evidence that subtle subjective cognitive concerns might be a harbinger of Alzheimer's disease-related cognitive decline.^15^, ^16^, ^17^, ^18^ In mutation carriers, greater subjective cognitive concerns were associated with accelerated long-term forgetting for two of the three components (list and story). This association is consistent with findings from previous epilepsy studies,^11^, ^12^, ^38^ although a study^39^ assessing a combination of different neurological diagnoses did not identify a relationship. Early subjective memory concerns in Alzheimer's disease, in the absence of conventional deficits, might reflect accelerated forgetting and impairments in long-term recall.

Our findings have parallels with a study of *APP* transgenic mice.^19^ Very young mice that had normal learning of a spatial navigation task and normal retention over a short interval had significantly reduced retention compared with wild-type mice when retested 7 days later. Furthermore, the deficit in long-term retention could potentially be rescued by anti-amyloid β immunotherapy.^19^ These results, alongside our findings, suggest that assessment of long-term forgetting in human beings might be useful as a marker of treatment response in presymptomatic therapeutic trials.

Our study has several limitations. First, the sample size is small, primarily because of the relative rarity of autosomal dominant Alzheimer's disease. Replication of our findings in both familial and sporadic presymptomatic Alzheimer's disease cohorts is therefore important. Although participants were asked not to discuss the testing following the initial learning and 30-min recall task, some individuals could have rehearsed the material during the intervening period, which is an inherent difficulty with accelerated long-term forgetting testing.^29^ However, when asked following the 7-day testing, all participants said that they had not rehearsed. Rehearsal might be more difficult to avoid in a trial with repeated measures, which could reduce test sensitivity. Therefore, it is important to identify methods to reduce the potential for rehearsal, such as embedding the accelerated long-term forgetting assessment material among other unrelated cognitive tests or making use of recognition tests for material that is difficult to rehearse.^40^, ^41^ We found a ceiling effect on the 7-day recognition testing, which could be reduced in future studies by using more demanding tests (eg, paired associate learning).^42^

We did not assess participants' sleep between initial learning and 7-day testing. Sleep is thought to play an important part in memory consolidation.^43^ However, a previous epilepsy study found sleep quality to have no direct effect on accelerated long-term forgetting,^44^ and we found no group difference in mood, which correlates with sleep.^45^ 7-day assessment was done remotely, which allows other sources of variability, including the surrounding environment, to add noise. Nevertheless, we detected group differences in retention at 7 days, suggesting robust effects.

We used parental age at onset to estimate when an individual would develop Alzheimer's disease symptoms. Although parental age at onset correlates closely with actual age at onset, it is a proxy measure,^7^ and only with longer-term follow-up can actual ages at symptom onset be confirmed.

In summary, we showed that accelerated long-term forgetting is present in autosomal dominant Alzheimer's disease mutation carriers, who were on average 7 years from predicted symptom onset, and thus might be a very early feature of Alzheimer's disease-related cognitive decline. Subtle increased subjective cognitive concerns also appear to be a presymptomatic feature of autosomal dominant Alzheimer's disease, which could be underpinned by accelerated long-term forgetting. Studies of accelerated long-term forgetting might provide insights into very early cognitive impairments in Alzheimer's disease, and accelerated long-term forgetting might be useful as part of inclusion criteria or as an outcome measure in presymptomatic Alzheimer's disease trials.

**This online publication has been corrected. The corrected version first appeared at thelancet.com/neurology on March 13, 2018**
