# Corrections

**DOI:** 10.1016/S1474-4422(18)30087-5

**Published:** 2018-04

*Weston P S J, Nicholas J M, Henley S M D, et al. Accelerated long-term forgetting in presymptomatic autosomal dominant Alzheimer's disease: a cross-sectional study.* Lancet Neurol *2018; **17:** 123–32*—In table 3 of this Article, data for figure 30-min recall were incorrectly recorded. Data in this row have been corrected in the online version as of March 13, 2018.

