# Supplementary material for: Accelerated long-term forgetting in presymptomatic autosomal dominant Alzheimer's disease: a cross-sectional study
Source: Lancet Neurol. 2018 Feb;17(2):123–32. doi: 10.1016/S1474-4422(17)30434-9 (PMC5958413; doi:10.1016/S1474-4422(17)30434-9)
Supplement: Supplementary appendix [file mmc1.pdf]

THE LANCET
Neurology

Supplementary appendix

This appendix formed part of the original submission and has been peer reviewed. We post it as supplied by the authors.

Supplement to: Weston PSJ, Nicholas JM, Henley SMD,et al. Accelerated long-term forgetting in presymptomatic autosomal dominant Alzheimer's disease: a cross-sectional study. *Lancet Neurol* 2018; **17:** 123–32.

Supplementary material 1: Table of participants' family mutations

The number of individuals from families with each mutation is given. Details relating to how many participants for each mutation were mutation carriers is not given to ensure it is not possible for any at risk individual to attempt to deduce their mutation status.

Gene	Mutation	Number of individuals
APP	p.Val717Asn	1
	p.Val717Ile	3
	p.Val717Leu	1
PS1	Intron 4	2
	Tyr115Cys	1
	p.Tyr115His	1
	p.Ser1132Ala	2
	p.Met139Val	2
	p.Met146Ile	3
	p.Glu184Asp	3
	p.Ile202Phe	4
	p.His214Thr	1
	p.Pro264Leu	1
	p.Arg278Ile	3
	p.Glu280Gly	6
	p.Ser290Cys	1

Supplementary material 2: Contents of envelope for seven-day phone assessment

- Blank A4 sheet (for figure recall).
- Pencil
- Figure recognition sheet, inside a separate envelope – participant asked not to open until the figure recall task is completed. The sheet displays four sets of three similar images, with one of each set of three being part of the original figure.
- Everyday memory questionnaire (EMQ)
- Stamped addressed envelope for return of figure recall and recognition assessments and EMQ
